# SERUM VITAMIN B12, IRON AND FOLIC ACID DEFICIENCIES IN OBESE INDIVIDUALS SUBMITTED TO DIFFERENT BARIATRIC TECHNIQUES

**DOI:** 10.1590/0102-6720201600S10016

**Published:** 2016

**Authors:** Rafaella de Andrade SILVA, Flávia Monteiro França MALTA, Maria Flora Ferreira Sampaio Carvalho CORREIA, Maria Goretti Pessoa de Araújo BURGOS

**Affiliations:** São João Hospital Center, E.P.E., Porto, Portugal

**Keywords:** Micronutrients, Vitamin B12, Iron, Folic acid, Bariatric surgery

## Abstract

**Background:**

Different surgical techniques to combat obesity combine malabsorption with restrictive procedures and can lead to metabolic problems, such as micronutrient deficiencies.

**Aim:**

Assess vitamin B12, iron and folic acid deficiencies associated with the lifestyle of obese individuals having been submitted to different bariatric techniques.

**Methods:**

A retrospective analysis was performed using the electronic charts of patients submitted to bariatric surgery involving adjustable gastric banding and Roux-en-Y gastric bypass at the São João Hospital Center in the city of Porto, Portugal, between 2005 and 2010. The following data were collected: surgical technique, sex, age, marital status, serum concentrations of vitamin B12, iron and folic acid and postoperative lifestyle. A 5% significance level was used for the statistical analysis (p<0.05).

**Results:**

Among 286 individuals evaluated, females accounted for 90.9% of the overall sample (both techniques). Gastric banding was performed more (68.9%), but greater nutrient deficiencies were found following gastric bypass. Iron was the most prevalent deficiency (21.3%), followed by vitamin B12 (16.9%) and folic acid (4.5%). Mild to moderate alcohol intake, adherence to the diet and the use of multivitamins reduced the frequency, but did not avoid micronutrient deficiency.

**Conclusion:**

Vitamin B12, iron and folic acid deficiencies were found in the first and second year following the two bariatric techniques analyzed and were more frequent among individuals submitted to gastric bypass.

## RESUMO

**Racional:**

As diferentes técnicas da cirurgia da obesidade combinam má absorção com procedimentos restritivos e podem levar à complicações metabólicas, entre as quais se destacam as deficiências de micronutrientes.

**Objetivo:**

Avaliar a deficiência de vitamina B12, ferro e ácido fólico e fatores associados ao estilo de vida de obesos submetidos a diferentes técnicas cirúrgicas.

**Métodos:**

Análise retrospectiva dos prontuários eletrônicos de pacientes submetidos à cirurgia bariátrica pelas técnicas de banda gástrica ajustável e bypass gástrico em Y-de-Roux, no Centro Hospitalar de São João, E.P.E., Porto - Portugal, no período de 2005-2010. Foram coletadas: técnica cirúrgica, sexo, idade, estado civil, concentrações séricas de vitamina B12, ferro e ácido fólico e o estilo de vida no pós-operatório. Para análise estatística foi utilizado nível de significância de 5% (p< 0,05).

**Resultados:**

Dentre os 286 indivíduos avaliados, houve predomínio do sexo feminino (90,9%) em ambas as técnicas cirúrgicas, sendo a banda gástrica a mais realizada (68,9%); no entanto maiores deficiências de micronutrientes foram detectadas após o bypass gástrico. A deficiência de micronutriente mais prevalente foi a de ferro (21,3%), seguida da vitamina B12 (16,9%) e do ácido fólico (4,5%). A ingestão de bebida alcoólica de leve-moderada, a adesão à dieta e o uso de polivitamínicos reduziu a frequência, mas não evitou a carência de micronutrientes.

**Conclusão:**

A deficiência de vitamina B12, ferro e ácido fólico foi observada durante o primeiro e o segundo anos após as duas técnicas avaliadas, sendo mais frequente nos pacientes submetidos ao bypass gástrico.

## INTRODUCTION

Obesity is a chronic, non-communicable disease with a multifactor etiology characterized by the excessive buildup of body fat and is related to an increase in the risk of mortality. Moreover, it is often accompanied by comorbidities, such as cardiovascular disease, dyslipidemia, systemic arterial hypertension, diabetes mellitus type 2 and some types of cancer^1^ ^,^ ^2^.

When clinical treatment (diet, physical exercise and medication) do not produce satisfactory results, bariatric surgery is an effective option that allows weight loss and the long-term maintenance of a stable body mass, along with a reduction in associated rates of comorbidities and mortality^1^ ^,^ ^3^. Different surgical techniques combine malabsorption with a restrictive technique and are associated with weight loss, but can also lead to metabolic complications, such as micronutrient deficiencies, especially with regard to vitamin B12, iron and folic acid^4^ ^,^ ^5^.

Considering the serious clinical repercussions of nutritional deficiencies in the short, medium and long terms, the aim of the present study was to evaluate deficiencies in the serum concentrations of vitamin B12, iron and folic acid as well as factors associated with lifestyle among obese individuals having been submitted to adjustable gastric banding or Roux-en-Y gastric bypass. 

## METHODS

This study received approval from the ethics committee of the São João Hospital Center in the city of Porto, Portugal. The non-use of a term of informed consent was authorized due to the fact that the data were collected from electronic clinical charts.

A retrospective study was conducted with information from the electronic records of patients having been submitted to bariatric surgery using either adjustable gastric banding or Roux-en-Y gastric bypass at the São João Hospital Center in Porto, Portugal. The inclusion criteria were age 20 years or older, having undergone preoperative and postoperative follow up by the same multidisciplinary team (surgeon, nutritionist, endocrinologist, psychiatrist and psychologist) and complete records of appointments up to two years following bariatric surgery between 2005 and 2010. The exclusion criteria were removal and/or replacement of the gastric band, having been submitted to further bariatric surgical techniques or other abdominal surgeries, having undergone plastic surgery in the preoperative or postoperative periods and pregnancy during any phase of the study. 

The following information was collected during the survey of the charts: surgical technique employed, gender, age, marital status, serum concentrations of vitamin B12, iron and folic acid as well as lifestyle in the postoperative period. Lifestyle was considered adequate when adherence to the diet proposed by the nutritionist occurred (≥75% compliance more than five days a week)^1^, when a daily multivitamin was taken (routine use of one capsule per day for >20 months of the 24-months evaluation period)^1^ and mild to moderate alcohol intake^6^. 

Deficiencies in serum concentrations of vitamin B12 and folic acid were analyzed through immunoassays using the electrochemiluminescence method with the aid of the automatic Elecsys equipment (Roche Diagnostics GmbH, Mannheim, Germany). Iron deficiency was measured based on the colorimetric method using a UV-Vis spectrophotometer (Thermo Scientific). The following cutoff points were employed: serum vitamin B12<187 pg/ml; serum folic acid <3.1 ng/ml; and serum iron <70 μg/dl for men and <60 μg/dl for women.

### Statistical analysis 

The data were digitized using the Microsoft Excel^TM^ 2010 program and the statistical analyses were performed with the aid of the Statistical Package for the Social Sciences, version 21 (SPSS Inc., Chicago, IL, USA). Descriptive statistics were performed (absolute and percentage frequencies). Inferential statistics involved either Pearson's chi-square test or Fisher's exact test, when appropriate. The margin of error used in the decisions of the statistical tests was 5% (p<0.05). 

## RESULTS

A total of 659 patients were submitted to bariatric surgery in the period studied (562 underwent gastric banding and 97 underwent gastric bypass). However, only 43.4% completed the two-year follow up and were included in the present study, resulting in a sample of 286 individuals.

Gastric banding accounted for 68.9% of the surgeries and the female sex was predominant (90.9%) in both types of surgery. No significant differences were found between the patients submitted to the different surgical techniques with regard to demographic or social variables (Table 1).

**TABLE 1:** Socio-demographic characteristics of patients submitted to gastric banding and gastric bypass; Porto, Portugal, 2005 to 2010

Variable	Type of surgery						
	Gastric banding		Gastric Bypass		Total Group		p
	n	%	n	%	n	%	
TOTAL	197	68.9%	89	31.1	286	100	
Sex							p(1) = 0.228
Female	178	90.4	82	92.1	260	90.9	
Male	19	9.6	7	7.9	26	9.1	
Age (years)							p(2) = 0.149
18 to 39	79	40.1	45	50.6	124	43.4	
40 to 64	115	58.4	44	49.4	159	55.6	
> 64	3	1.5	-	-	3	1.0	
Marital status							p(2) = 0.960
Single	34	17.3	15	16.9	49	17.1	
Married	152	77.2	68	76.4	220	76.9	
Widowed	2	1.0	1	1.1	3	1.0	
Divorced	9	4.6	5	5.6	14	4.9	

(1) Pearson's chi-square test. (2) Fisher's exact test

The prevalence of vitamin B12, iron and folic acid deficiencies was higher among the patients submitted to gastric bypass than those submitted to gastric banding. The differences were significant in the first year following gastric bypass with regard to folic acid, in the second year with regard to iron and throughout the entire two-year follow-up period with regard to vitamin B12 (Table 2). 

**TABLE 2:**
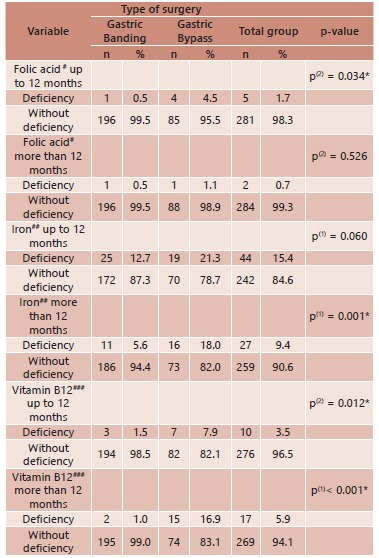
Deficiencies in serum concentrations of vitamin B12, iron and folic acid according to type of bariatric surgery and postoperative period; Porto, Portugal, 2005 to 2010

(*) Significant difference at 5.0% level. (1) Pearson's chi-square test. (2) Fisher's exact test. Cutoff points: #< 3.1 ng/mL; ##men: < 70 μg/dl, women: <60 μg/dl; ###< 187 pg/ml

No significant association was found between the reduction in the concentrations of vitamin B12, iron or folic acid and adherence to diet, the use of a daily multivitamin or mild to moderate alcohol intake (Table 3).

**TABLE 3:**
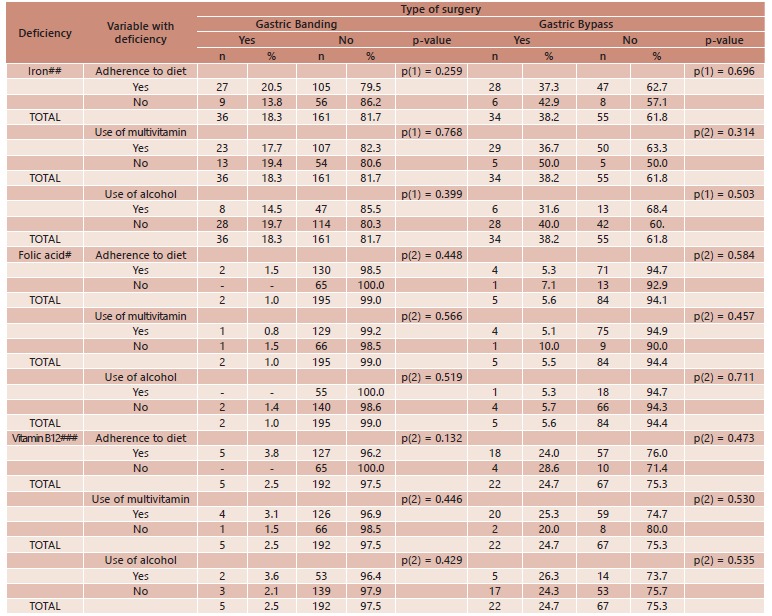
Associations between iron, folic acid and vitamin B12 deficiencies and adherence to diet, use of multivitamins and alcohol intake according to type of bariatric surgery; Porto, Portugal, 2005 to 2010

(1) Pearson's chi-square test; (2) Fisher's exact test. Cutoff points: #< 3.1 ng/mL; ##men: < 70 μg/dl, women: <60 μg/dl; ###< 187 pg/ml

 The correlation between serum concentrations of vitamin B12, iron and folic acid did not achieve statistical significance, except between iron and vitamin B12 deficiencies in the first year following gastric banding, suggesting that these nutritional deficiencies did not occur concomitantly (Table 4). No associations were found between iron deficiency and either age or gender with either type of bariatric surgery (Table 5).

**TABLE 4:**
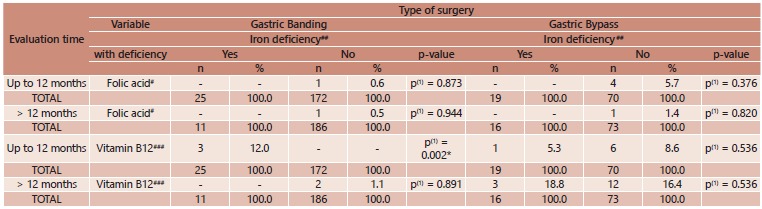
Correlation between iron deficiency and both folic acid and vitamin B12 deficiency according to postoperative period and type of bariatric surgery; Porto, Portugal, 2005 to 2010

(*) Significant difference at 5.0% level, (1) Fisher's exact test, (2) Pearson's chi-square test, Cutoff points: ^#^< 3.1 ng/mL; ^##^men: < 70 μg/dl, women: <60 μg/dl; ^###^< 187 pg/ml

**TABLE 5:**
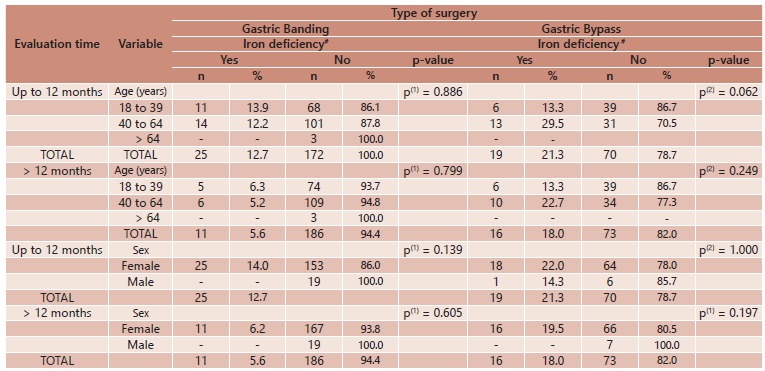
Association between iron deficiency and both age and gender according to evaluation time and type of bariatric surgery; Porto, Portugal, 2005 to 2010

(1) Fisher's exact test. (2) Pearson's chi-square test

## DISCUSSION

The predominance of the female and the 40-to-64-year-old age group in the present study is in agreement with data described by Karefylakis,^7^ who found that 85% of the 431 individuals submitted to gastric bypass surgery were women and mean age was 51.3 ± 10 years. Likewise, Alvarez^8^ found that 75% of patients submitted to vertical gastrectomy were women and mean age was 40±10 years. In the present study, the majority of the sample was married, which is in agreement with data described by Amorim^9^ who found that 65.5% of the patients were either married or in a stable union. It is therefore plausible that women in different populations are more concerned with health and esthetics than men during the ageing process and in romantic relationships^9^. 

The frequency of reductions in serum concentrations of vitamin B12, iron and folic acid was lower among the patients submitted to gastric banding, which was the more common technique employed in the sample analyzed in comparison to gastric bypass. This finding is in disagreement with data reported in previous investigations that evaluated the occurrence of anemia and micronutrient deficiencies following gastric bypass alone^4^ ^,^ ^7^ or in combination with vertical gastrectomy^5^ ^,^ ^8^. 

Scientific evidence suggests that nutritional deficiencies, especially with regard to vitamins and minerals, are common in candidates for bariatric surgery in the preoperative period^10^. In the postoperative period, Carvalho^4^ found that 23.1% of patients had reduced serum vitamin B12 levels, with this figure increasing to 47.2% six months following gastric bypass surgery. The reduction in serum concentrations of vitamin B12 in the present study was significantly greater in both the first and second years following gastric bypass surgery. Acidity and peptic hydrolysis help release vitamin B12 from foods so that this vitamin couples to the intrinsic factor (released by parietal cells) and the intrinsic factor-vitamin B12 complex is absorbed in the terminal ileum^4^. Achlorhydria, reduced vitamin B12 intake due to intolerance to foods that are the main source of this vitamin (meat and milk) and a reduction in the secretion of the intrinsic factor necessary for its absorption are possible aspects that contribute to the high prevalence of this deficiency^11^.

Inadequate serum concentrations of folic acid were rare in comparison to iron and vitamin B12 in the present sample, which is similar to data described by Vargas-Ruiz^12^ who report that folic acid deficiency was not found in any patient and that iron and vitamin B12 deficiencies (iron deficiency anemia and megaloblastic anemia, respectively) were more frequent. Nutritional deficits following bariatric surgery may also be explained by the lack of a balanced diet fin the postoperative period. Such patients have excessive body fat due to the inadequate consumption of foods that are rich in carbohydrates and fats, especially cholesterol, trans fats and saturated fats, while also poor in important nutrients, such as vitamins, minerals and fibers, thereby favoring the occurrence of nutritional deficiencies in these individuals^10^. 

Iron deficiency anemia and megaloblastic anemia have been described as unavoidable in patients submitted to bariatric surgery who are not treated in a prophylactic manner^13^. Adherence to diet and the use of multivitamins were found 72.4% and 73.1% of the patients studied, respectively (data not presented in tables), which can be considered satisfactory, but did not avoid deficiency of the micronutrients evaluated. Patients tend to resist the adoption of new eating habits following bariatric surgery when they do not attend appointments with the multidisciplinary team with due diligence. The high frequency of the use of a multivitamin in the present investigation is in divergence with results reported by Karefylakis^7^, who found that only 23.9% of the sample took a multivitamin. 

The use of an alcoholic beverage (mainly wine) by only 25.9% of the sample (data not presented in a table) is a lower frequency than that reported for other populations, possibly due to the fact that the majority of the sample was made up of women. However, alcohol intake tends to be higher in other studies^9^. Replacing the excessive consumption of food with alcohol is a common practice following bariatric surgery due to the satisfaction generated following its ingestion in some individuals^6^. 

Anemia is common following bariatric surgery, but atypical causes, such as tumors, should be suspected in older patients and especially individuals who are refractory to clinical control^13^. According to Karefylakis^7^, anemia seems not to progress with the postoperative time following bariatric surgery and is less prevalent in patients with regular medical examinations, which underscores the importance of the long-term follow up of such patients.

No correlations were found among the serum concentrations of vitamin B12, iron and folic acid, except between iron deficiency anemia and megaloblastic anemia in the first year following gastric banding, which suggests that these nutritional deficiencies occurred in an isolated fashion. Although women are considered to be a group at risk for iron deficiency anemia due to monthly blood loss, no association was found in the present study between deficiency in the serum concentration of iron and either age or sex for either of the types of bariatric surgery analyzed.

## CONCLUSION

Iron deficiency anemia and megaloblastic anemia were more frequent than folic acid deficiency in the sample of patients analyzed. Moreover, deficiencies of these micronutrients were more common among the patients submitted to Roux-en-Y gastric bypass than those submitted to adjustable gastric banding. Adherence to diet and the use of a multivitamin reduced the frequency of such deficiencies, but did not impede some degree of iron, vitamin B12 and folic acid deficiency. 

## References

[1] Mechanick JI, Youdim A, Jones DB (2013). Clinical practice guidelines for the perioperative nutritional,
metabolic and nonsurgical support of the bariatric surgery patient. Surg Obes Relat Dis.

[2] Tobias DK, Pan A, Jackson CL (2014). Body-Mass Index and Mortality among Adults with Incident Type 2
Diabetes. N Engl J Med.

[3] Parri A, Benaiges D, Schröder H (2015). Preoperative predictors of weight loss at 4 years following bariatric
surgery. Nutr Clin Pract.

[4] Carvalho IR, Loscalzo IT, Freitas MFB, Jordão RE, Friano TC. (2012). Incidência da deficiência de vitamina B12 em pacientes submetidos à
cirurgia bariátrica pela técnica Fobicapella (Y-de-Roux).. ABCD Arq Bras Cir Dig.

[5] Kwon Y, Kim HJ, Lo Menzo E, Park S, Szomstein S, Rosenthal RJ (2014). Anemia, iron and vitamin B12 deficiencies after sleeve gastrectomy
compared to Roux-en-Y gastric bypass a meta-analysis. Surg Obes Relat Dis.

[6] Burgos MG, Cabral PC, Maio R, Oliveira BM, Dias MS, Melim DB, Correia MF (2015). Prevalence of Alcohol Abuse Before and After Bariatric Surgery
Associated With Nutritional and Lifestyle Factors A Study Involving a Portuguese
Population. Obes Surg.

[7] Karefylakis C, Näslund I, Edholm D, Sundbom M, Karlsson FA, Rask E (2015). Prevalence of anemia and related deficiencies 10 years after gastric
bypass--a retrospective study. Obes Surg.

[8] Alvarez V, Cuevas A, Olivos C, Marcos B, Farías MM (2014). Déficit de micronutrientes a más de un año de postoperatorio en
gastrectomía en manga. Nutrición Hospitalaria.

[9] Amorim ACR, Souza AFO, Nascimento ALV, Maio R, Burgos MGPA (2015). Uso de bebida alcóolica em períodos pré e pós-operatório de cirurgia
bariátrica. Rev. Col. Bras. Cir.

[10] Lima KVG, Costa MJC, Gonçalves MCR, Sousa BS (2013). Deficiências de micronutrientes no pré-operatório de cirurgia
bariátrica. ABCD Arq Bras Cir Dig.

[11] Shah M, Simha V, Garg A (2006). Review Long-Term Impact of Bariatric Surgery on body weight,
comorbidities, and nutritional status. J Clin Endocrinol Metab.

[12] Vargas-Ruiz AG, Hernández-Rivera G, Herrera MF (2008). Prevalence of iron, folate, and vitamin B12 deficiency anemia after
laparoscopic Roux-en-Y gastric bypass. Obes Surg.

[13] Baretta GAP, Marchesini JB, Marchesini JCD, Brenner S, Sanches MER (2008). Anemia pós-cirurgia bariátrica as causas nem sempre são relacionadas à
cirurgia. ABCD Arq Bras Cir Dig.

